# MiR-155 promotes anaplastic thyroid cancer progression by directly targeting SOCS1

**DOI:** 10.1186/s12885-019-6319-4

**Published:** 2019-11-12

**Authors:** Wei Zhang, Wenyue Ji, Xudong Zhao

**Affiliations:** 1 Department of Endocrinology, Shengjing Hospital, China Medical University, Shenyang, 110004 China; 2 Department of Otorhinolaryngology Shengjing Hospital, China Medical University, No. 36 Sanhao Street, Heping District, Shenyang, 110004 China

**Keywords:** miR-155, SOCS1, Anaplastic thyroid cancer, ATC

## Abstract

**Background:**

Anaplastic thyroid cancer (ATC) is considered to be a rare type of thyroid cancer but takes up the most important proportion of thyroid cancer-related deaths. Therefore, the development of molecular targeted therapy is an exciting strategy in the management of ATC.

**Methods:**

miR-155 and SOCS1 expression were measured by qRT-PCR as well as western blot analysis. 8305c and FRO cells were transfected and cultured for apoptosis assays, transwell, MTT on miR-155 or SOCS1 suppression and overexpression. Dual-luciferase reporter assays and SOCS1 restoration experimentswas implemented for define the relation between SOCS1 and miR-155. In addition, the correlation between miR-155 expression and patients’ clinicopathological features were also explored.

**Results:**

Aberrant miR-155 and SOCS1 expression and inverse correlation were found in ATC samples. In addition, it indicated that miR-155 expression correlated with cervical metastasis as well as extrathyroidal invasion. Moreover, we demonstrated that miR-155 inhibited 8305c and FRO cells apoptosis, promoted proliferation, invasion and migration. Furthermore, miR-155 inhibition was associated with a significant overexpression of SOCS1. Additionally, luciferase reporter assays presented that miR-155 could bind to SOCS1 3′-UTR, influencing its stability negatively and finally lowering SOCS1 levels. Moreover, it was illustrated that the impacts of miR-155 suppression were reversed by the inhibition of SOCS1 on cell proliferation, apoptosis as well as invasion.

**Conclusions:**

Aberrant miR-155/SOCS1 expression has been included in ATC progression: miR-155 overexpression leads to SOCS1 suppression and develops ATC progression. Thus, miR-155 has been considered to be an underlying therapeutic target for ATC.

## Background

Thyroid cancer is considered to be the most common endocrine tumor [1]. Most of the thyroid cancers are well-differentiated, and patients are usually cured by surgical resection. Anaplastic thyroid cancer (ATC) is considered to be the rare subtype of thyroid cancer [2] but takes up the important proportion of thyroid cancer-related deaths [3, 4]. Currently, no effective treatments are available for ATC patients. Therefore, the development of molecular targeted therapy may be an exciting method in managing ATC. MicroRNAs (miRNAs) are small and non-protein-coding RNA which regulate genes expression through binding to the target mRNA’s 3′ untranslated region [5]. MiR-155 is reported to develop tumor growth as well as invasion in a lot of solid malignancies, like breast [6–9], colon [10–12], gastric [13, 14], liver [15, 16] and oral [17] cancers. Meanwhile, the potential mechanism and function of miR-155 in ATC are still not clear. SOCS1 is a member of the suppressor of cytokine signaling (SOCS) family, proteins which perform as cytokine signal transduction’s negative regulator [18]. Our previous study indicated that miR-155 can promote the migration and invasion of laryngeal cancer through targeting SOCS1 [19]. In addition, Xue et al. defined the tumor-promoting function of miR-155/SOCS1 pathway in lung cancer progression [20]. Also, Merkel et al. found that miR-155 downregulated SOCS1 to promote anaplastic large cell lymphoma [21]. The function of SOCS1 in ATC is not yet known. The potential mechanism and impact of miR-155/SOCS1 in ATC was determined in this study. We also validated the regulatory relation between SOCS1 and miR-155 in ATCs. The miR-155 might be an effective marker for predicting prognosis.

## Methods

### Study subjects and patient tissue samples

A total of 31 paired ATC and adjacent thyroid tissue samples were obtained from patients that experienced surgery in the Shengjing Hospital of China Medical University from 2013 to 2016. The research had been supported by the Ethics Committee of Shengjing Hospital. Relevant clinical information was gathered from patients’ records and informed written consent had been gained. All the samples (including cancer and normal thyroid tissue) are diagnosed by three pathologists to confirm the histologic diagnosis.

### Quantitative real-time polymerase chain reaction (qRT-PCR)

The overall RNA was extracted by adopting TRIzol reagent. The miRNA expression was implemented by adopting the TaqMan miRNA assay. The related quantification of Gene expression was normalized through the 2^−ΔΔCt^ strategy related to *U6*. Every experiment was carried on in triplicate.

### Western blotting

Total protein of tissues and cells was separated by electrophoresis on 8% polyacrylamide gels and then electrotransferred to polyvinylidene fluoride (PVDF) membranes. After that, the PVDF membranes was incubated with primary anti-SOCS1 (12,000, Abcam, Cambridge, MA, USA) as well as ß-actin (12,000, Abcam, MA, USA) antibody at 4 °C for a night, followed by 2-h incubation with a secondary antibody. Image J software was adopted for quantifying the integrated density of the bands.

### Transient transfection and cell culture

The human ATC cell lines 8305c (Cat NO. SCSP540) and FRO (Cat NO. SCSP577) were obtained from Cell bank of typical culture preservation Committee of Chinese Academy of Sciences. 8305c cell line was originally derived from anaplastic thyroid carcinoma of an adult female. FRO cell line was also originally derived from anaplastic thyroid carcinoma of an adult female. These cell lines have been authenticated by using Single Tandem Repeat (STR) profiling method. There is no mycoplasma contamination in 8305c and FRO cell lines. 8305c cells were cultured within MEM (the medium of the modified Eagle) supplemented in a humidified 5% CO2 incubator with 10% fetal bovine serum (FBS) at room temperature. FRO cells were cultured within DMEM (the medium of the modified Eagle) with 10% fetal bovine serum (FBS). MiR-155 ASO, mimic and negative controls were bought from Genecopoeia. Empty vector (EV) as well as SOCS1-specific siRNA and overexpression vector were gained from Shanghai Genechem Co., LTD. Cells were transfected by adopting the Lipofectamine 2000 kit (Invitrogen) in accordance with the instructions of the manufacturer.

### Cell proliferation assays

Counting Kit-8 (CCK-8) (Invitrogen) was used to evaluate the cell proliferation ability. Cells was seeded at a density of 2000 cells/100 μL within 96-well plates and the absorbance was calculated in an ELx-800 Universal Microplate Reader (BioTek Instruments, Winooski, VT, USA) at 450 nmat 0, 24, 48, 72 and 96 h.

### Cell invasion assays

Cell invasion assays were carried out by transwell chambers coated with Matrigel (BD Biosciences, Bedford, MA, USA). Briefly, 5 × 10^4^ cells in 200 μL of medium were placed with an 8-μm pore size polycarbonate filter in the upper chamber (BD Biosciences). The lower chamber was added with culture medium containing 10% FBS. The cells which invaded the lower chamber membranes was stained with 0.1% crystal violet after being incubating for two whole days(Merck, Darmstadt, Germany) for 30 min, and photographed under a microscope (Olympus, Tokyo, Japan).

### Dual-luciferase reporter assay

Luciferase assays were implemented by adopting the luciferase reporter assay system in accordance with the instructions of the manufacturer. The mutated (Mut) or wild-type (WT) SOCS1 3′-UTR sequence including the miR-155 targeting site was inserted into pGL3 (Invitrogen) for the purpose of constructing pGL3-luc-SOCS1. 8305c and FRO cells were transfected with pGL3-luc-SOCS1 (50 ng) and seeded in 96-well plates, miR-155 negative or mimic control (5 pmol) as well as renilla luciferase (5 ng) by adopting Lipofectamine 2000 (Invitrogen). The dual luciferase assay detected the luciferase activity after two whole days (Promega).

### Apoptosis assays

8305c and FRO cells were harvested after transfection at 48 h, and 7-aminoactinomycin Das well as annexin-V (7-AAd) antibody was added, followed by incubation at room temperature. After 15 min, flow cytometry assay was implemented through adopting an LSR II flow cytometer (BD Biosciences). The outcomes were discussed by adopting Flow Jo software. Annexin V as well as 7-AAd double positive cells was defined to be apoptotic cells. 7-AAd negative as well as annexin V positive cells were regarded to be in the primary apoptosis procedure.

### Colorimetric caspase-3 assays

8305c and FRO cells were lysed primarily, and then protein concentration had been decided. An overall of 100 μg protein was incubated for two hours by using 10 μL of Ac-DEVD-pNA (Abcam, America) at room temperature, and the absorbance at 405 nm was calculated by adopting a microplate reader (BioTek Instruments).

### Statistical analysis

Information was shown to be mean ± SD and discussed by adopting the student t-test. A paired t-test was adopted for paired samples. Pearson test was used to define the relationship between miR-155 and SOCS1. Statistical analyses were conducted with SPSS 17.0 (SPSS Inc., USA) software. *P* < 0.05 was regarded statistically important.

## Results

### Overexpression of miR-155 in human ATC tissues

**Fig. 1:**
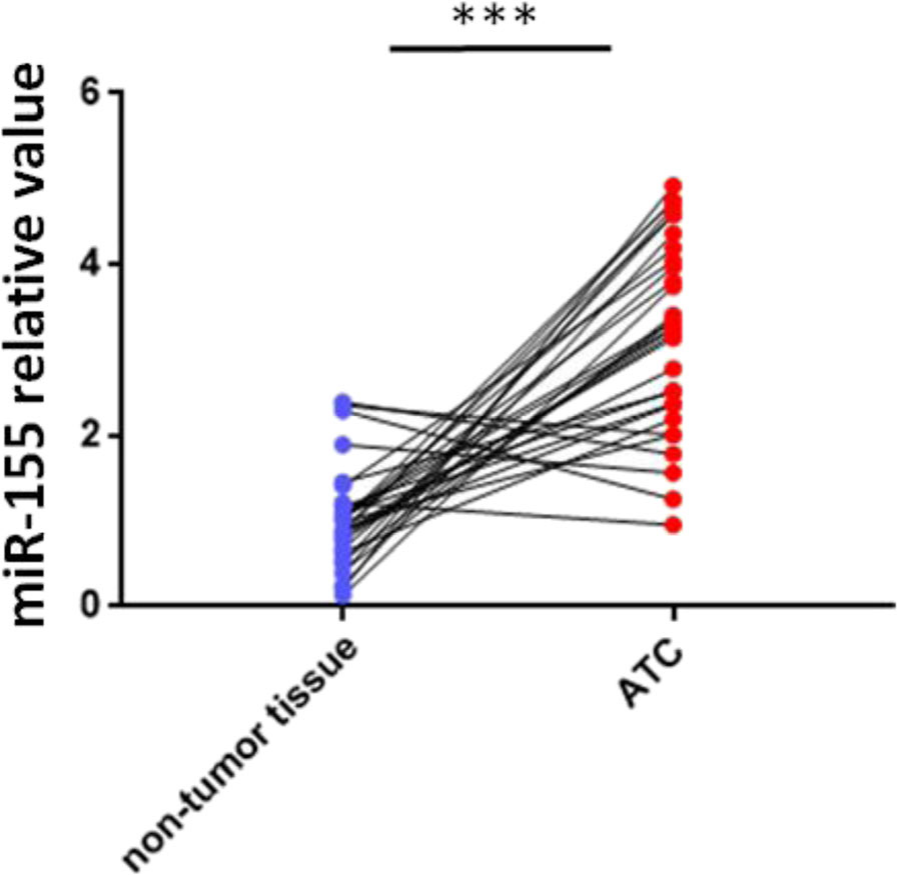
Overexpression of miR-155 in ATC organized system. Performing PCR analysis during the quantification process of miR-155 deliverance in 31 paired ATC and adjacent non-tumor tissues. Twenty-six ATC specimens had higher miR-155 expression compared with non-tumor tissues. Research on log transformed data by t-test (*P* < 0.001). Every experiment has to be done three times. *U6* was used for normalization

Adjacent non-tumor tissue samples as well as 31 paired ATC were tested by qRT-PCR for the purpose of determining the expression of miR-155 in ATCs. By comparing with the adjacent non-tumor tissue samples, 26 ATC specimens were found to have higher miR-155 expression (Fig. 1).

### MiR-155 may promote ATC progression and metastasis in humans

Then, the correlation between miR-155 expression and clinicopathological parameters was analyzed. The outcomes for miR-155 were presented in Table 1. No correlation was discovered between miR-155 expression and multicentricity, tumor size, sex or age. Nevertheless, higher miR-155 expression in patients with cervical lymph node metastasis and with extrathyroidal invasion were found. In conclusion, the outcomes indicate miR-155 is possible to promote the metastasis as well as ATC progression.

**Table 1:** Correlation of miR-155 expression with clinicopathological factors of ATC patients

Parameters	Patients	miR-155	*P*
Total	31		
Gender			0.942
Male	5 (16.1)	3.46 ± 1.52	
Female	26 (83.9)	3.15 ± 1.06	
Age (Y)			0.513
≥ 45	16 (51.6)	3.34 ± 1.18	
< 45	15 (48.4)	3.10 ± 1.07	
Tumor size,cm			0.204
≥ 2	25 (80.6)	3.36 ± 1.20	
< 2	6 (19.4)	2.71 ± 0.98	
Extrathyroidal invasion			0.033
Yes	24 (77.4)	3.78 ± 1.24	
No	7 (22.6)	1.47 ± 0.51	
Multicentricity			0.142
Yes	17 (54.8)	3.41 ± 1.21	
No	14 (45.2)	2.95 ± 1.01	
Cervical metastasis status			0.021
N+	22 (44.7)	4.16 ± 1.38	
N-	9 (55.3)	1.35 ± 0.47	

Note: Values in parentheses represent percentages

### The forced suppression of MiR-155 expression inhibits the migration and proliferation of cells and enhances the apoptosis of ATC cells in vitro

**Fig. 2:**
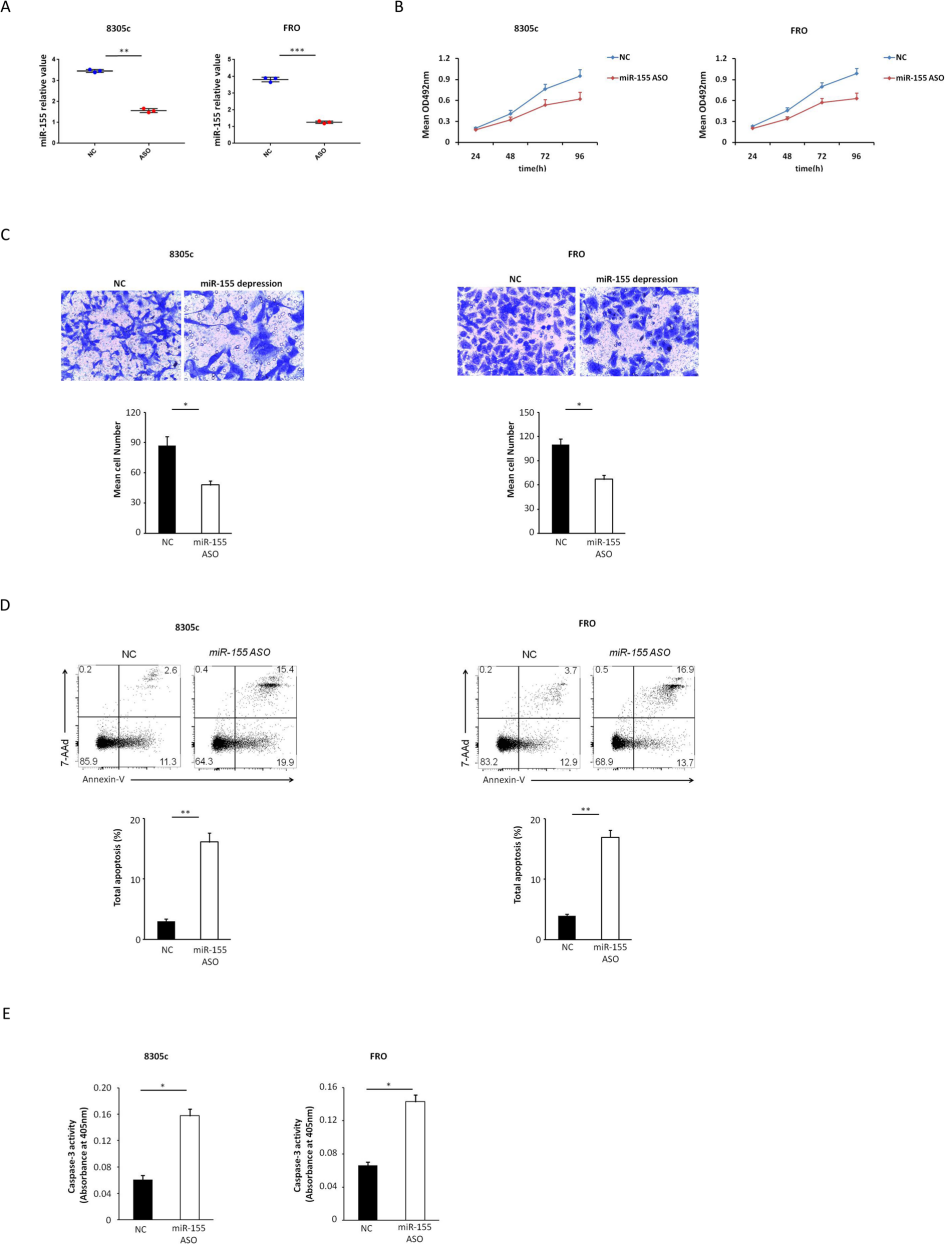
Influence of decreased miR-155 on 8305c and FRO cell proliferation, invasion, and apoptosis. **a** 8305c and FRO cells were instantaneous transfection with miR-155 ASO or negative control (8305c: 1.57 ± 0.11 vs. 3.46 ± 0.09 [control], *P* = 0.005; FRO: 1.26 ± 0.04 vs. 3.82 ± 0.08 [control], P < 0.001). **b** Cell survival rates in MTT assay in miR-155-suppressed and control 8305c and FRO cells. The rate of increase in cell number decreased in miR-155-suppressed cells was found (8305c: 11.7% ± 2.1, 21.7% ± 2.6, 20.0% ± 3.2 and 34.9% ± 3.9 at 24, 48, 72, and 96 h, respectively, *P* = 0.037; FRO: 12.9% ± 1.7, 25.8% ± 2.3, 28.5% ± 3.6 and 36.3% ± 4.2 at 24, 48, 72, and 96 h, separately, *P* = 0.023). **c**Typical photo (upper; zoom in 200 times) and fixed quantity discuss (bottom) of determination of Transwell mobile and aggressive attacks in miR-155-suppressed and negative dominate 8305c and FRO cells. **d** Typical photo and rate of aging death of miR-155-suppressed or negative control 8305c and FRO cells (**P = 0.009 and P = 0.005 for 8305c and FRO cells, respectively). **e** Caspase-3 activity in miR-155-suppressed or negative control 8305c and FRO cells (*P = 0.031 and *P* = 0.044 for 8305c and FRO cells, respectively). All trials are done three times

MTT assays were adopted for evaluating the impact of miR-155 suppression on the proliferation of 8305c and FRO cells. We transfected transiently 8305c and FRO cells with miR-155 ASO or negative control (Fig. 2a). It was discovered that the forced suppression of miR-155 inhibited the development of 8305c and FRO cells compared with negative control in a time-dependent manner (Fig. 2b). The transwell assay was adopted for determining migratory potential of 8305c and FRO cells with miR-155 suppression. Compared with negative control, forced suppression of miR-155 resulted in decreased 8305c and FRO cell invasion (*P* = 0.027 and 0.041) (Fig. 2c). Annexin-V staining was implemented for determining the impact of miR-155 suppression on cell apoptosis. 8305c and FRO cells with miR-155 suppression greatly enhanced the apoptosis (Fig. 2d). Similar results were obtained with the colorimetric caspase 3 assay (Fig. 2e). Collectively, these data indicated that the inhibition of miR-155 expression reduces the invasion and proliferation of ATC cells and enhances apoptosis in vitro.

### Overexpression MiR-155 promotes the migration and proliferation of ATC cells in vitro

**Fig. 3:**
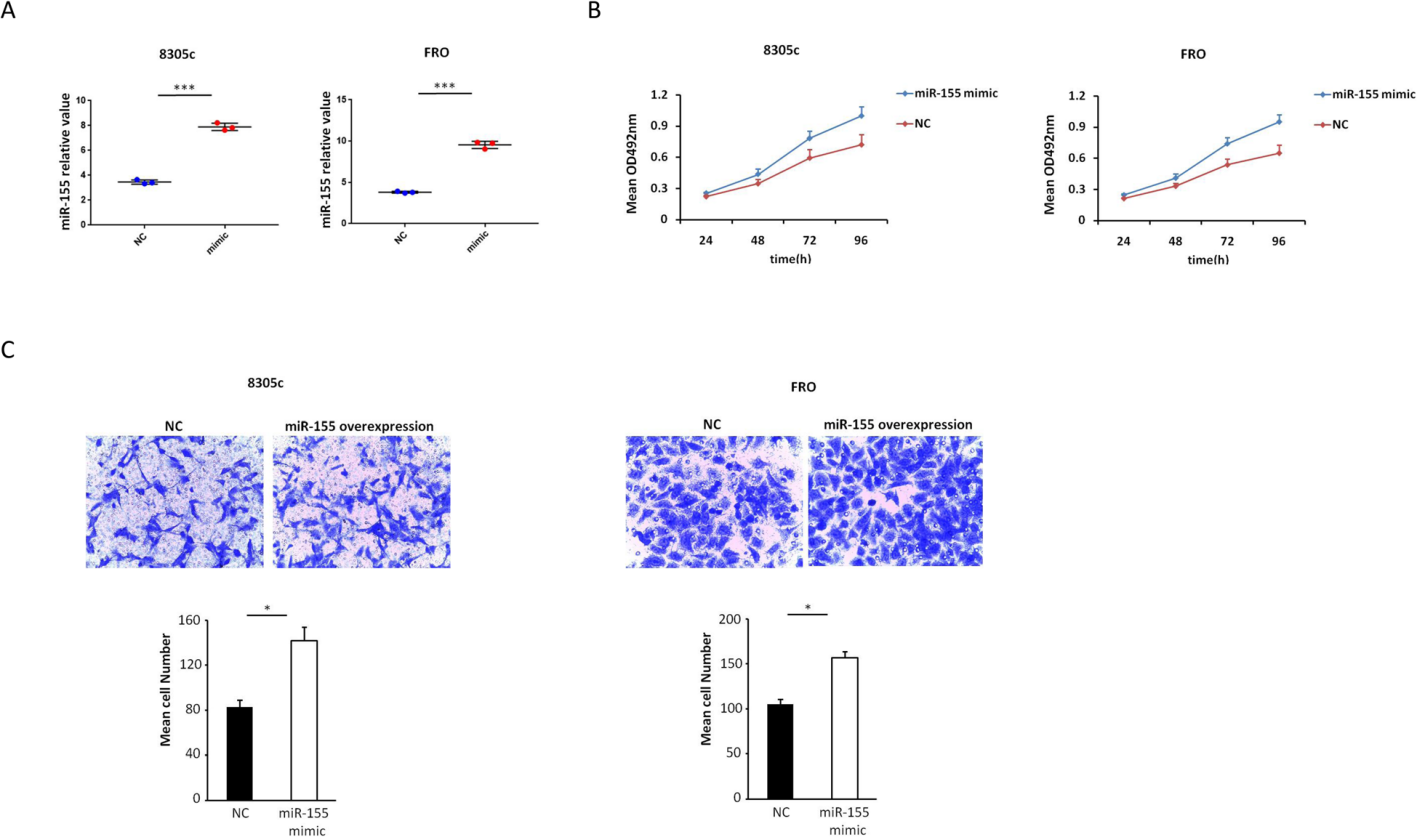
Influence of increased miR-155 on 8305c and FRO cell proliferation and invasion. **a** 8305c and FRO cells were instantaneous transfection with miR-155 mimic or negative control (8305c: 7.90 ± 0.17 vs. 3.47 ± 0.10 [control], *P* < 0.001; FRO: 9.54 ± 0.25 vs. 3.81 ± 0.06 [control], *P* < 0.001). **b** Cell survival rates in MTT assay in miR-155 overexpression and control 8305c and FRO cells. The rate of increase in cell number increased in miR-155 overexpression cells was found (8305c: 13.4% ± 1.2, 25.6% ± 2.1, 31.5% ± 3.6 and 38.4% ± 4.4 at 24, 48, 72, and 96 h, respectively, *P* = 0.044; FRO: FRO: 17.1% ± 2.4, 24.2% ± 2.7, 38.4% ± 4.1 and 46.7% ± 5.3 at 24, 48, 72, and 96 h, separately, *P* = 0.041). **c**Typical photo (upper; zoom in 200 times) and fixed quantity discuss (bottom) of determination of Transwell mobile and aggressive attacks in mimic overexpression and negative dominate 8305c and FRO cells (8305c, *P* = 0.033; FRO: *P* = 0.048). All trials are done three times

MTT and transwell assays were used to evaluate the effect of miR-155 overexpression on the proliferation and migration of 8305c and FRO cells. We transfected transiently 8305c and FRO cells with miR-155 mimic or negative control (Fig. 3a). We found that upregulation of miR-155 promoted the proliferation of 8305c and FRO cells compared with negative control in a time-dependent manner (Fig. 3b). Also, compared with negative control, overexpression of miR-155 resulted in increased ATC cell invasion (Fig. 3c). Collectively, these data indicated that overexpression of miR-155 promotes the invasion and proliferation of ATC cells.

### MiR-155 directly inhibits SOCS1 expression in vitro

**Fig. 4:**
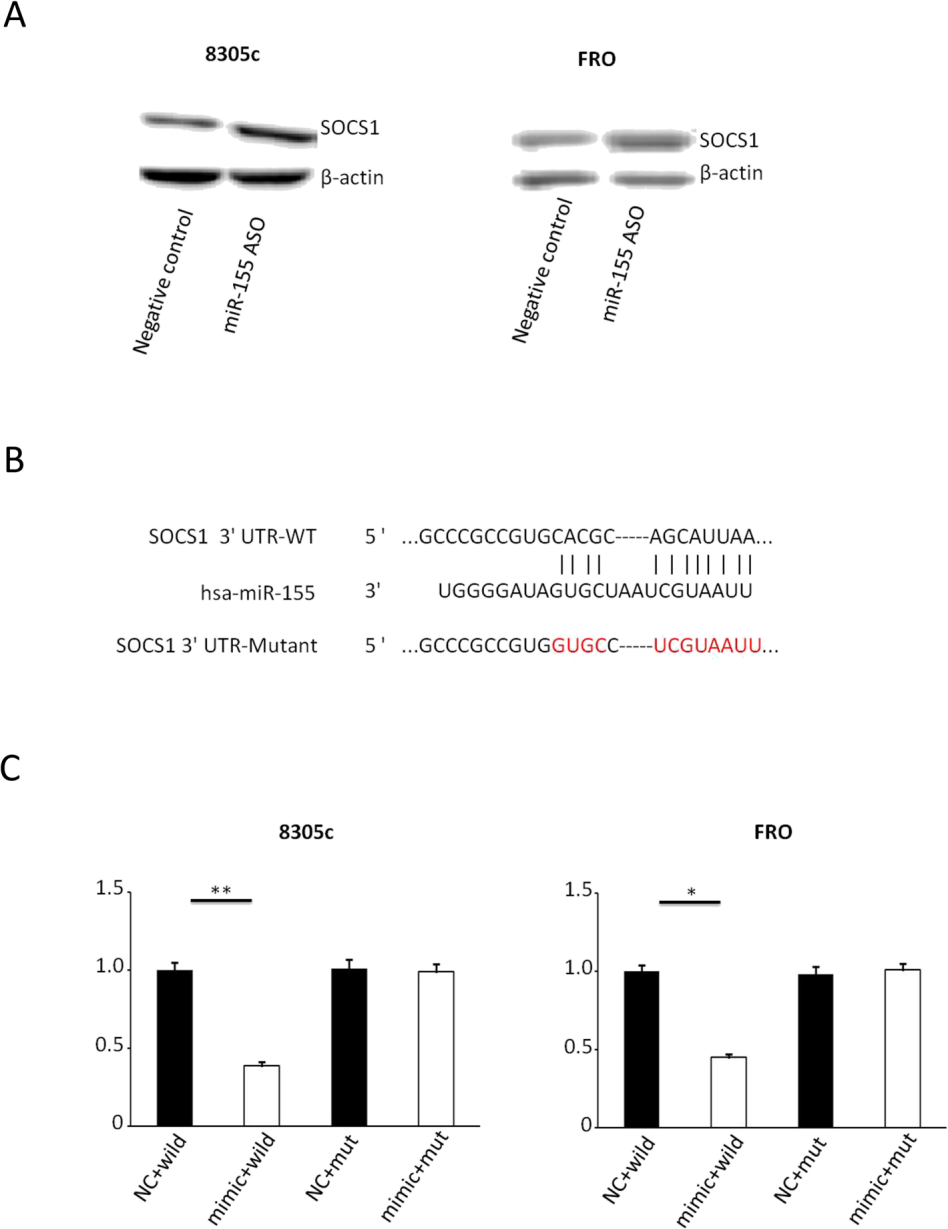
SOCS1 is a direct target of miR-155 in ATC. **a** Western special blot and number of SOCS1 protein expression in 8305c and FRO cells infection with miR-155 mimic or passive bridle (8305c: 81.45 ± 7.11 vs. 41.55 ± 3.17, *P* = 0.033; FRO: 90.33 ± 5.51 vs. 52.13 ± 2.98, P = 0.048). Using β-actin as a reference. **b** miR-155 diametrically interplay with the 3′-UTRs of SOCS1. **c** The relative luciferase activity of the reporter with natural growth-type SOCS1 3′-UTR was significantly decreased after transfection with miR-155 mimic. Nevertheless, after the mutation of 3′-UTR with SOCS1, the luciferase activity of the reporter was not affected by miR-155. The data obtained is the average ± SD. The log transformed data was analyzed using two-way analysis of variance. **P = 0.008 and *P* = 0.017 for 8305c and FRO cells, respectively

SOCS1 was predicted to be the underlying target of miR-155 by bioinformatics analysis (PicTar, TargetScan and miRWalk). Then, 8305c and FRO cells were transfected with miR-155 ASO. The miR-155 suppression was linked to an important increase in the expression of SOCS1 by comparing with the negative control (Fig. 4a). Moreover, we performed luciferase reporter assays for the purpose of determining the relation between SOCS1 and miR-155. Luciferase reporter plasmids including either the mutated 3′-UTR or the wild-type of SOCS1 were constructed (Fig. 4b). The reporter’s related luciferase activity including the wild-type SOCS1 3′-UTR greatly reduced on its co-transfection with miR-155 mimic. Nevertheless, the reporter’s luciferase activity including the mutant 3′-UTR wasn’t affected (Fig. 4c). The outcomes implied SOCS1 was the direct target of miR-155 in ATC.

### Downregulation of SOCS1 in human ATC tissues

**Fig. 5:**
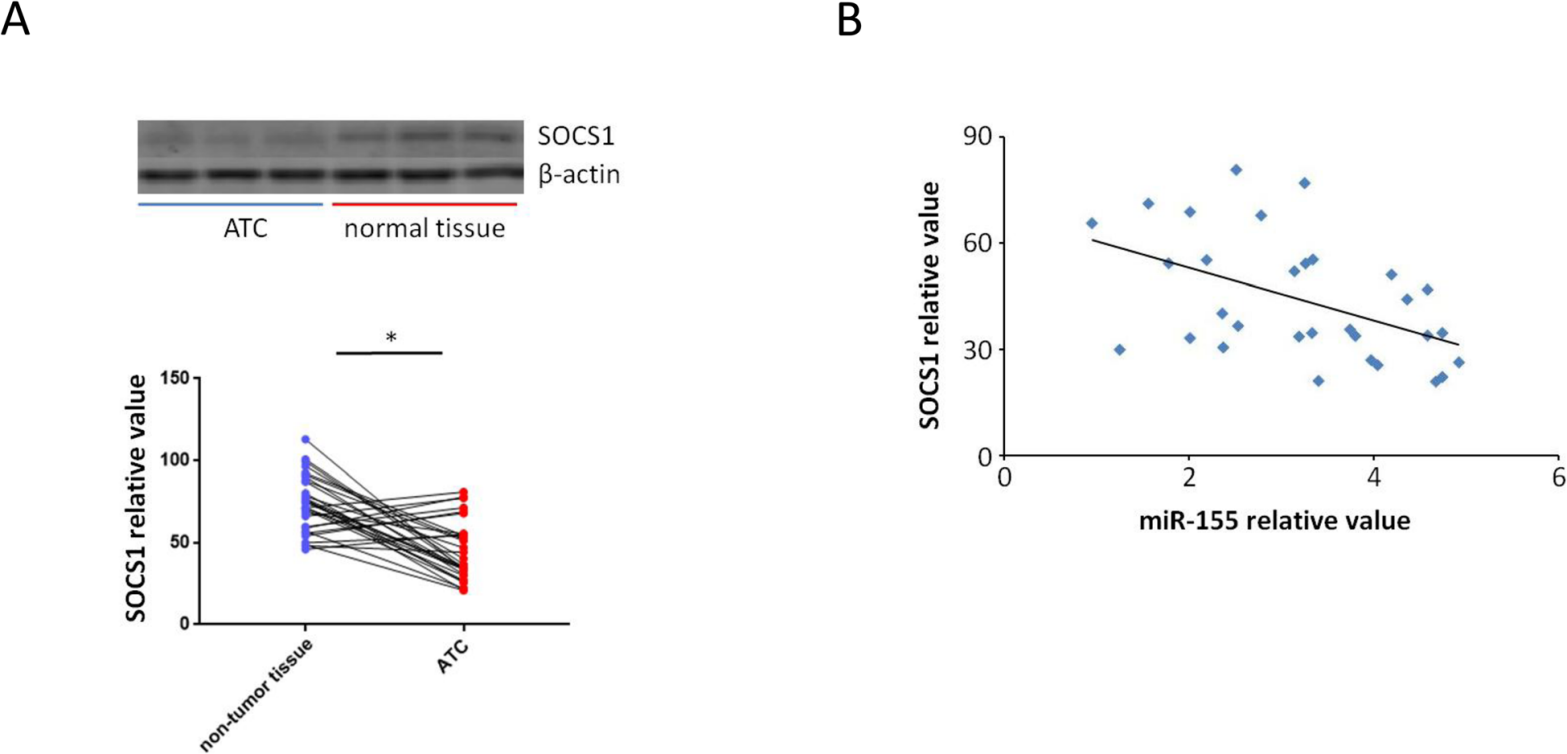
Downregulation of SOCS1 in ATC samples. **a** Western blot were used to test 31 paired ATC and adjacent non-tumor tissue samples. 24 ATC specimens were found to have lower SOCS1 protein expression (*P* = 0.039). Every experiment has to be done three times. Using β-actin as a reference. **b** Pearson test was used to determine the relationship between miR-155 and SOCS1 expression. Inverse correlation was found between them (Pearson correlation coefficient was − 0.48, *P* = 0.006)

Adjacent non-tumor tissue samples as well as 31 paired ATC were tested by western blot for the purpose of determining the expression of SOCS1 in ATCs. By comparing with the adjacent non-tumor tissue samples, 24 ATC specimens were found to have lower SOCS1 protein expression (Fig. 5a). Furthermore, Pearson test was used to determine the relationship between miR-155 and SOCS1 expression. Negative correlation was found between them (Fig. 5b).

### Upregulation of SOCS1 expression inhibits the migration and proliferation of ATC cells in vitro

**Fig. 6:**
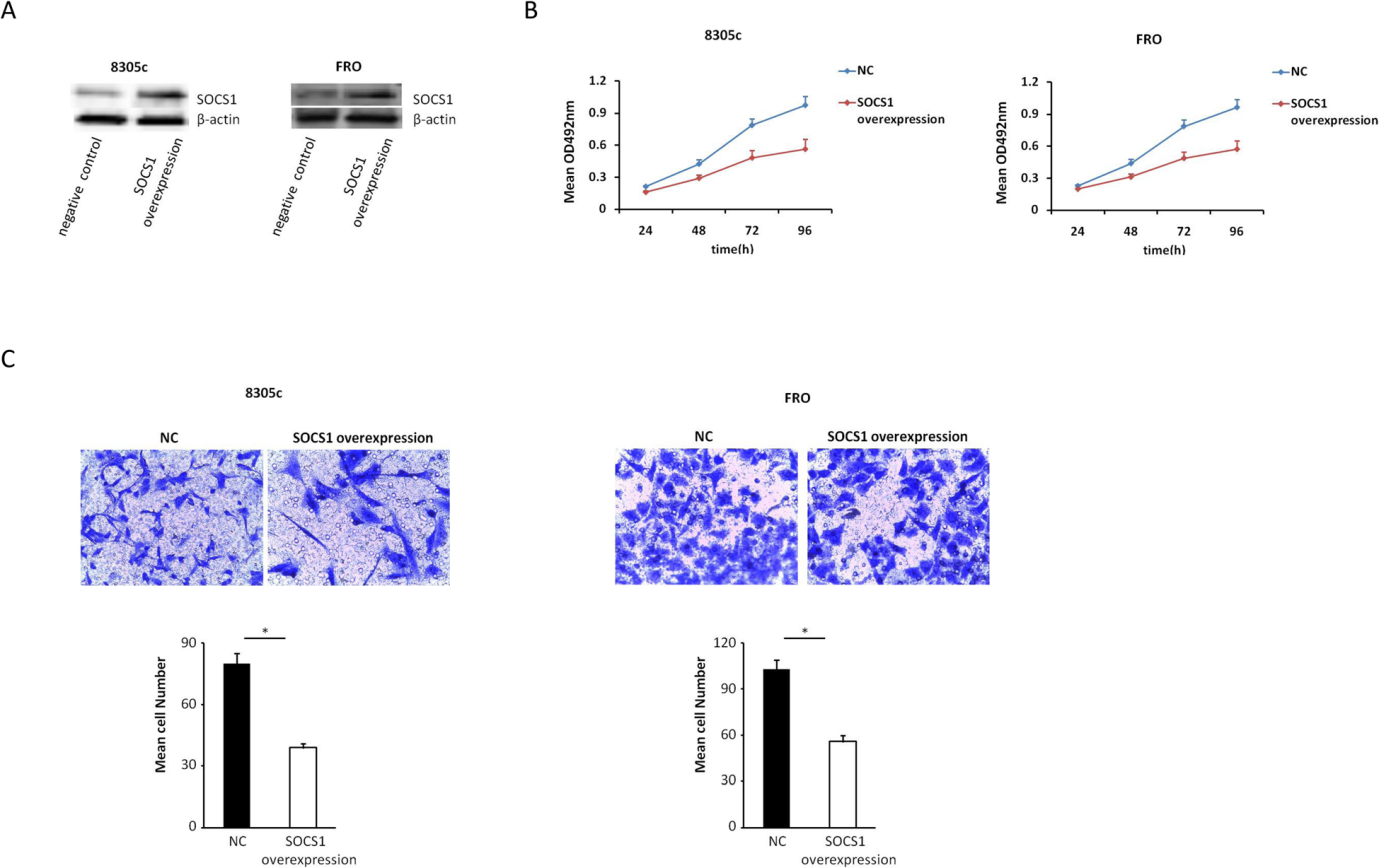
Effect of SOCS1 on 8305c and FRO cell proliferation and invasion. **a** 8305c and FRO cells were instantaneous transfected with SOCS1-overexpressing vector or negative control (8305c: 85.76 ± 5.22 vs. 42.33 ± 3.72 [control], *P* = 0.003; FRO: 93.41 ± 5.79 vs. 51.76 ± 3.21 [control], *P* < 0.001). **b** Cell survival rates in MTT assay in SOCS1 overexpression and control 8305c and FRO cells. The rate of increase in cell number decreased in SOCS1 overexpression cells was found (8305c: 23.6% ± 2.2, 31.1% ± 3.5, 38.7% ± 3.9 and 42.2% ± 5.7 at 24, 48, 72, and 96 h, respectively, *P* = 0.0013; FRO: 13.5% ± 1.5, 29.5% ± 3.1, 38.1% ± 4.6 and 40.8% ± 6.1 at 24, 48, 72, and 96 h, separately, *P* = 0.019). **c** Typical photo (upper; zoom in 200 times) and fixed quantity discuss (bottom) of determination of Transwell mobile and aggressive attacks in SOCS1 overexpression and negative dominate 8305c and FRO cells (8305c, *P* = 0.019; FRO: *P* = 0.036). All trials are done three times

MTT and transwell assays were adopted for evaluating the impact of SOCS1 overexpression on the proliferation and migration of 8305c and FRO cells. We transfected transiently 8305c and FRO cells with SOCS1-overexpressing vector or negative control (Fig. 6a). We found that upregulation of SOCS1expression inhibited the development of 8305c and FRO cells compared with negative control in a time-dependent manner (Fig. 6b). Also, compared with negative control, upregulation of SOCS1 resulted in decreased ATC cell invasion (Fig. 6c). Collectively, these data indicated that upregulation of SOCS1 reduces the invasion and proliferation of ATC cells.

### SOCS1 knockdown reverses the impacts of miR-155 silencing

**Fig. 7:**
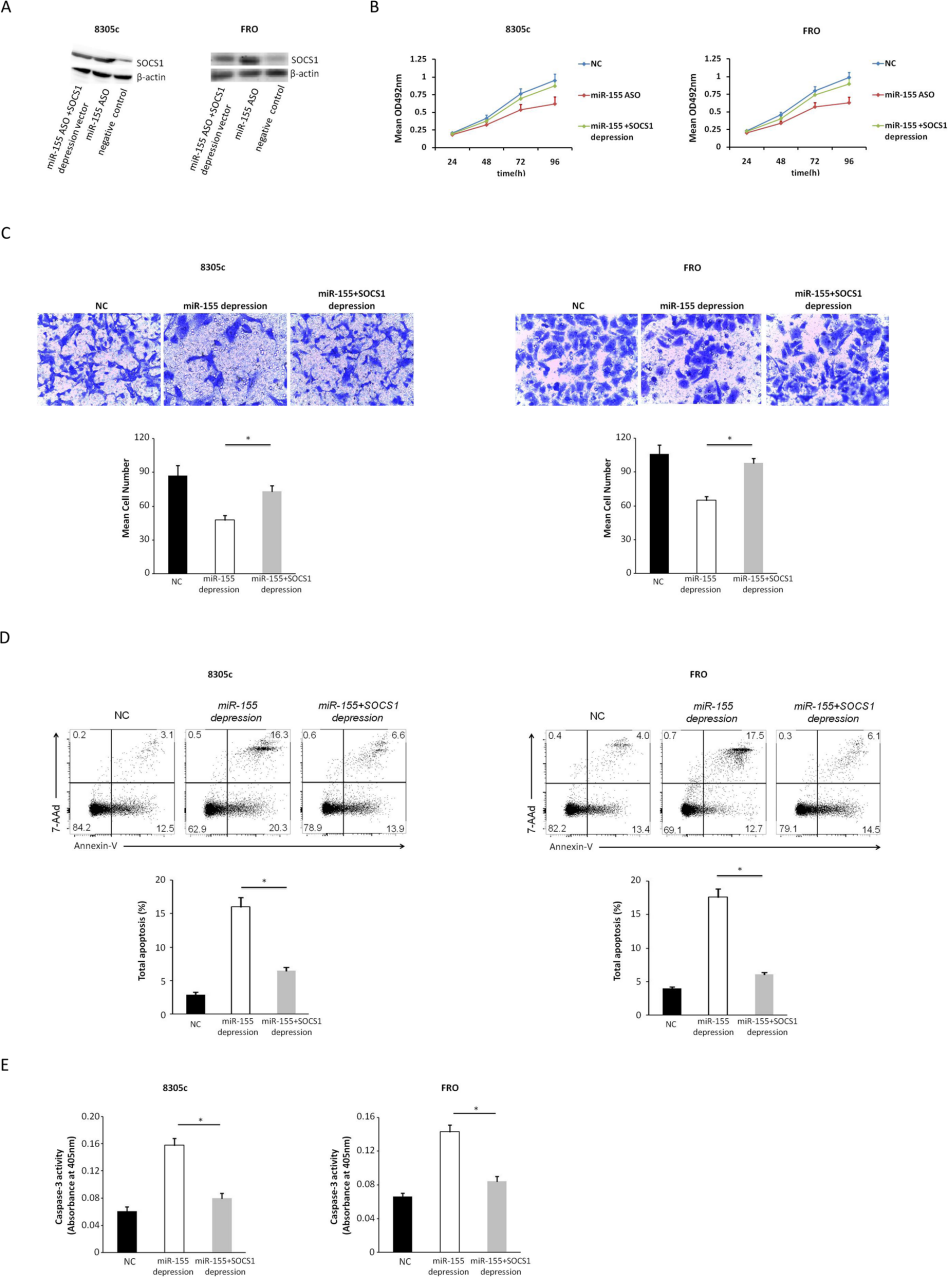
SOCS1 knockdown reverses the effects of miR-155. (NC: negative control). **a** Representative western blot showing the silencing of SOCS1 in 8305c and FRO cells after co-infection of miR-155 ASO and SOCS1-specific siRNA compared with cells transfected with miR-155 ASO independent. **b** Increase in the number of miR-155-silenced 8305c and FRO cells at different points in time after SOCS1 knockdown. **c** Typical photo (upper; zoom in 200 times) and fixed quantity discss (bottom) of transwell assays in 8305c and FRO cells transfection with miR-155 ASO have and not have SOCS1 knockdown (*P = 0.032 and *P* = 0.046 for 8305c and FRO cells, respectively). **d** Typical photo of the ratio of 8305c and FRO cells apoptosis after transfection with miR-155 ASO have and not have SOCS1 knockdown (*P = 0.037 and *P* = 0.021 for 8305c and FRO cells, respectively). **e** Caspase-3 of 8305c and FRO cells transfected with miR-155 ASO with and without SOCS1 knockdown (*P = 0.028 and P = 0.046 for 8305c and FRO cells, respectively)

SOCS1-specific siRNA was transfected into miR-155-silenced 8305c and FRO cells (Fig. 7a) for the purpose of further understanding SOCS1-mediated impacts of miR-155, restoring SOCS1 expression (8305c:42.01 ± 3.62 vs. 73.42 ± 6.13 in cells transfected with ASO alone, *P* = 0.047; FRO:44.12 ± 3.11 vs. 85.48 ± 4.79 in cells transfected with ASO alone, *P* = 0.027), and found that SOCS1 knockdown restored the promotion effects of miR-155 on cell invasion as well as proliferation within miR-155-silenced cells (Fig. 7b and c). By comparing with miR-155-silenced 8305c and FRO cells, 8305c and FRO cells with SOCS1 knockdown presented a time-dependent increase in the proliferation of cells (8305c: 7.7 ± 0.3, 16.8 ± 2.1, 29.8 ± 3.7, and 41.2 ± 4.1% at 24, 48, 72, and 96 h, respectively, *P* = 0.045; FRO: 11.4 ± 0.4, 18.3 ± 1.7, 29.3 ± 3.1, and 42.6 ± 3.8% at 24, 48, 72, and 96 h, respectively, *P* = 0.033). Furthermore, SOCS1 knockdown greatly inhibited apoptosis (Fig. 7d and e). The information indicates miR-155 promotes ATC progression by inhibiting SOCS1.

## Discussion

Thyroid cancer is considered to be the most ordinary malignancy of endocrine system, influencing over 3.2 million people all over the world [22]. Follicular thyroid cancer as well as papillary thyroid cancer takes up about 90% of all the thyroid malignancies. Nevertheless, the most lethal histotype is ATC, where the related survival rates with five years are just about 8% [23]. Thus, it’s of great necessary to deeply understand the molecular foundation of ATC etiology. MiR-155 is reported to develop a lot of progression and improvement of solid malignancies. Zuo et al. highlighted that miR-155 develops the progression of breast cancer [7], while Qu et al. claimed miR-155 increased cell proliferation, metastasis as well as invasionin colorectal carcinoma [10]. Also, Sun et al. presented the cell invasion and development was accelerated by miR-155 in the gastric carcinoma cells in humans [14]. Furthermore, Zhang et al. revealed that miR-155 promotes tumor growth in human hepatocellular carcinoma [15]. Moreover, several studies have found that miR-155 develops oral squamous cell carcinoma cell proliferation [17, 18]. The over-expression of miR-155 in 31 paired ATC tissues was discovered in the current research. The correlation between clinicopathological parameters and miR-155 was also analyzed and discovered extrathyroidal invasion and cervical metastasis correlated with higher miR-155. Moreover, 8305c and FRO cells were adopted for investigating the impact of miR-155 aberrant expression within ATC cells. It was illustrated miR-155 improved 8305c and FRO cells proliferation, invasion as well as migration, and inhibited apoptosis. Thus, it could be summarized miR-155 promoted the progression of ATC. In addition, for the purpose of determining the regulatory mechanisms of miR-155 in ATC progression, miRanda, PicTar and Targetscan were adopted for predicting the targets of miR-155. SOCS1 was discovered as an underlying target for miR-155. SOCS1 is a member of the SOCS family. Our previous study showed that SOCS1 inhibited the laryngeal cancer’s migration and invasion through targeting Stat3 [19]. Also, Xue et al. determined the inhibitory function of SOCS1in non-small cell lung cancer progression [20]. Furthermore, Merkel et al. found that miR-155 downregulated SOCS1 to promote anaplastic large cell lymphoma [21]. In the current research, SOCS1 was found to be significantly decreased and inverse correlated with miR-155 in ATC samples. Also, 8305c and FRO cells were adopted for investigating the relation between miR-155 and SOCS1. Deregulation of miR-155 had been connected with an important increase in the degrees of SOCS1. Additionally, luciferase reporter assays presented miR-155 was able to SOCS1 3′-UTR, influencing negatively its stability and finally lowering the SOCS1 level. Moreover, it is proved the deregulation of SOCS1 reversed the impacts of the inhibition of miR-155 expression on cell apoptosis, invasion as well as proliferation. The investigation of tumorigenicity assays in nude mice might further shed light on the role of SOCS1 and miR-155 in ATC progression as well as the probability to adopt them as targets for the clinical applications in the future.

## Conclusions

Our study indicated that aberrant miR-155 expression was contained in ATC progression. miR-155 overexpression promotes cancer progression via SOCS1 deregulation. Therefore, miR-155 is a potential therapeutic target for ATC.

## Data Availability

The datasets used or analysed during the current study are available from the corresponding author on reasonable request.

## Abbreviations

ATC: Anaplastic thyroid cancer
CCK-8: Cell Counting Kit-8
FBS: Fetal bovine serum
MEM: Medium of the modified Eagle
miRNAs: microRNAs
NC: Negative control
PVDF: Polyvinylidene fluoride
qRT-PCR: Quantitative real-time polymerase chain reaction
SOCS: Suppressor protein of cytokine signaling
STR: Single Tandem Repeat
WT: Wild-type
